# Conductive Polymer Foaming: A Review on Fundamentals, Technology and Applications

**DOI:** 10.3390/polym18091043

**Published:** 2026-04-25

**Authors:** Xin Hu, Xiaodong Luo, Gang Wang, Mengyao Dong, Li Zhou, Xin Pan, Meiling Du, Xiangning Zhang, Kun Li, Xiaoli Zhang, Jingbo Chen

**Affiliations:** 1 School of Mechanical and Intelligent Manufacturing, Chongqing University of Science and Technology, Chongqing 401331, China; 2024202057@cqust.edu.cn; 2 Key Laboratory of Material Processing and Mold Technology, School of Mechanical Engineering, Chongqing Industry Polytechnic University, Chongqing 401120, China; wanggang@cqipu.edu.cn (G.W.); dongmy@cqipu.edu.cn (M.D.); zhouli@cqipu.edu.cn (L.Z.); panxin@cqipu.edu.cn (X.P.); duml@cqipu.edu.cn (M.D.); xiangningzhang@cqipu.edu.cn (X.Z.); 3 School of Materials Science and Engineering, Zhengzhou University, Zhengzhou 450001, China; zhangxl@zzu.edu.cn (X.Z.); chenjb@zzu.edu.cn (J.C.)

**Keywords:** conductive polymers, microcellular foaming, electromagnetic shielding, flexible sensors, thermal management

## Abstract

Conductive polymer microcellular foamed materials are a type of functional composite that combines lightweight cell structures with controllable conductivity. Their core feature lies in regulating the cell structure of the material through microcellular foaming technology, along with the introduction of conductive fillers or the intrinsic conductivity of the polymer, to achieve enhanced electrical performance. This paper systematically reviews conductive polymers and their microcellular foamed materials, highlighting research progress in foaming mechanisms, preparation processes, and functional applications. It first analyzes the key mechanisms of bubble nucleation, growth, and stabilization during the microcellular foaming of conductive polymers. Then, it elaborates on the research status and functional mechanisms of these materials in three typical application scenarios: electromagnetic shielding, flexible sensors, and thermal management. Finally, it outlines the future development directions of conductive polymer microcellular foamed materials in multifunctional integration, green fabrication, and intelligent applications, aiming to provide theoretical guidance and technical pathways for future research.

## 1. Introduction

With the rapid development of electronic technologies, the market demand for materials that combine electrical conductivity with mechanical flexibility has increased significantly. Traditional conductive materials, despite exhibiting favorable electrical conductivity, are inherently heavy and rigid, rendering them ill-suited to meet the stringent requirements of portable and flexible electronic devices. The advent of conductive polymers has addressed this gap by presenting a promising class of materials that not only maintain robust conductive properties but also possess exceptional flexibility and processability. Nevertheless, notwithstanding their well-established molecular-scale conductive networks, the practical utility of conductive polymers remains constrained in high-performance applications such as aerospace and wearable devices fields that demand ultra-lightweight materials [1,2,3,4,5]. To address this limitation, conductive polymer microcellular foaming technology has emerged as an effective mitigation strategy through the introduction of microcellular architecture. This approach confers dual advantages: first, the microcellular structure enhances the conductive network while concurrently reducing material density, thereby maintaining relatively stable electrical conductivity; second, the multiple interfaces formed by the microcellular framework can improve electromagnetic shielding effectiveness (EMI SE) [6,7,8]. Furthermore, sensors fabricated from such foamed materials demonstrate notable performance metrics, including high sensitivity, superior compressive strength and a broad operational range [9,10]. The influence of conductive polymer microcellular foaming on thermal management performance is a multifaceted process encompassing multi-scale interactions and diverse mechanistic pathways. Its effects extend beyond optimizing traditional insulation/conduction functions, to enabling the intelligent and multifunctional integration of thermal management systems via structural innovation [11,12].

Conductive polymer microcellular foams are typically fabricated through a process involving the dissolution of a physical foaming agent within a conductive polymer matrix, followed by specialized processing techniques. The formation of the cellular structure is primarily governed by the foaming agent reaching a supersaturated state within the system, induced by abrupt pressure reduction or temperature elevation, which triggers thermodynamic instability. This instability promotes pore nucleation and growth, as dissolved gas is liberated from the conductive polymer/gas mixture, ultimately culminating in the formation of a cell architecture [13,14]. Traditionally, conductive polymer foams have been produced using chemical foaming techniques; however, to meet the increasing demand for environmentally benign, sustainable, and pollution-free processes, supercritical fluids such as CO_2_ and N_2_ have emerged as promising green alternatives to conventional chemical blowing agents. Among them, supercritical CO_2_ has gained the most extensive application and demonstrates the highest potential for future development in supercritical foaming technology [15,16,17,18,19,20,21,22].

In recent years, several review articles have systematically summarized polymer foaming and supercritical fluid-assisted processing from different perspectives. For example, Wang mainly focused on the foaming behavior of polypropylene (PP) under supercritical CO_2_ conditions, including the effects of chemical and crystalline structures, reinforcement strategies, foaming technologies, and applications [23]. Dong and David emphasized the physicochemical characteristics of supercritical fluids, their dissolution and diffusion behavior in polymers, and their general roles in foaming and other polymer-processing operations [24,25]. Ritima discussed the factors governing the foamability of thermoplastic materials and the strategies used to improve foaming performance [26], whereas Chimezie reviewed the progress and limitations in preparing low-density nanocellular foams through batch foaming, extrusion foaming, and injection foaming [27]. Collectively, these reviews have provided an important basis for understanding the thermodynamics, kinetics, structural regulation, and processing technologies of polymer foaming. These works have provided an important foundation for understanding polymer foaming mechanisms, processing methods, and structural regulation. However, dedicated reviews on conductive polymer microcellular foams as a distinct class of functional materials are relatively few. In particular, the coupling among conductive-network construction, cellular-structure evolution, and functional performance has been less frequent in discussions, especially in representative applications such as electromagnetic interference shielding, flexible sensing, and thermal management. Therefore, this article presents a comprehensive review of conductive polymer microcellular foams, focusing on their conductivity mechanisms, foaming dynamics, processing technologies, and applications. Initially, we classify conductive polymers based on their conductivity mechanisms and analyze the key factors influencing their electrical performance. Subsequently, we examine the microcellular foaming mechanisms of these materials and elaborate on associated foam-forming processes. Finally, we explore the diverse application areas of conductive polymer foams, highlighting their potential in advancing next-generation electronic and functional devices. Compared with previous reviews, this work specifically emphasizes the intrinsic linkage among foaming mechanisms, conductive-network formation, and function-oriented applications, thereby aiming to provide a clearer framework for the rational design of lightweight and multifunctional conductive foams.

## 2. Conductive Polymers

Conductive polymer materials represent a novel class of functional materials that integrate polymer characteristics with electrical conductivity [28]. Based on distinct conduction mechanisms, these materials are primarily categorized into two classes: structural conductive polymers and composite conductive polymers. Notably, composite conductive polymers have emerged as the dominant type in industrial applications, owing to their well-established fabrication methodologies, cost-effectiveness, and the ability to tune electrical conductivity via filler incorporation [29]. In contrast, despite their unique molecular conductivity mechanisms, such as conjugated π-electron delocalization, structural conductive polymers remain largely confined to laboratory research settings. This limitation stems from several inherent challenges, including poor structural stability, difficulties in processability and formability, and suboptimal overall mechanical performance [30].

In recent years, advancements in material synthesis methodologies have enabled researchers to substantially enhance the electrical conductivity of composite conductive polymers through strategies including polymer blending, conductive filler doping, and multi-phase composite design. Particularly, within the domain of microcellular foaming, the synergistic optimization of cell architecture and conductive networks has proven transformative: this approach not only reduces material density but also preserves or even augments electrical conductivity. Such progress has thus paved the way for the development of lightweight, highly conductive, and multifunctionally integrated materials [31].

### 2.1. Structural Conductive Polymer Materials

Structured conductive polymer materials encompass conjugated polymers that exhibit intrinsic electrical conductivity by virtue of their molecular architecture, as well as polymers that attain conductivity through intentional doping processes. Their conductivity mechanisms are primarily governed by soliton theory and energy band theory [32,33,34]. The electrical conductivity of these materials is jointly modulated by both the doping mechanism and process parameters [35,36,37,38,39,40]. For instance, Akhil K. Poddar [41] outlined the polymerization techniques for synthesizing conducting polymers (CPs) and also reported the electrical conductivity values of common conducting polymers in both doped and undoped states. Sun [42] investigated the multifaceted roles of dopants in conductive polymers, demonstrating how conductivity regulation occurs through temperature-dependent adjustments in molecular chain arrangement, charge carrier mobility, and dopant stability. Similarly, Chiang [43] examined the influence of temperature on the charge transport properties of doped polyacetylene, elucidating the temperature-dependent nature of the metal-insulator transition. The primary preparation methods for structured conductive polymer materials include chemical polymerization [44] and electrochemical polymerization [45]. Furthermore, modified preparation techniques, such as gas-phase polymerization and solution polymerization, have been developed [46,47]. Notably, distinct preparation methodologies exert significant influences on the structural integrity, molecular weight and electrical conductivity of these polymers [48,49].

### 2.2. Composite Conductive Polymer Materials

Conductive polymer composites (CPC) are defined as multiphase composite systems endowed with conductive functionality, fabricated through the incorporation of conductive fillers into a polymer matrix, followed by processing techniques such as dispersion, lamination, surface modification, or gradient composite treatment. These composites uniquely integrate the high electrical conductivity of the conductive fillers with the mechanical flexibility and processability inherent to the polymer matrix [50].

**Figure 1:**
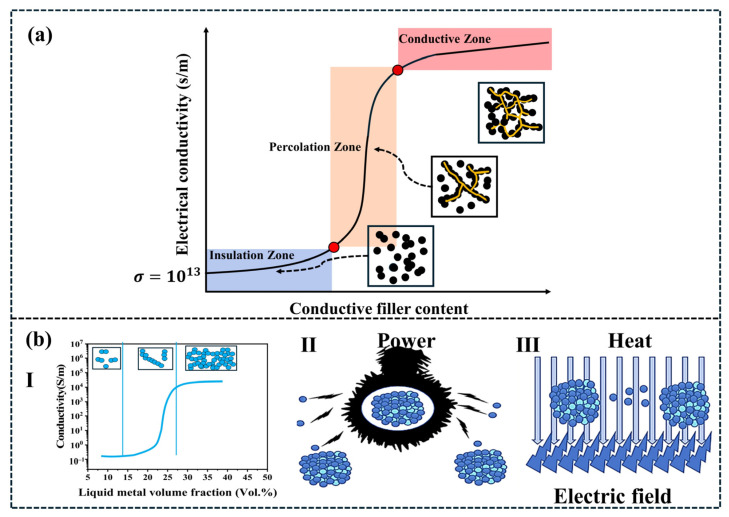
(**a**) Conductivity of filled CPCs vs. addition of conductive materials [29]. (**b**) Schematic of conductive mechanism. (**I**) Conduction path theory. (**II**) Electron tunneling theory. (**III**) Field emission theory [30].

The conductivity mechanism of conductive polymer composites (CPCs) is inherently intricate, broadly categorized into two key dimensions: the formation of conductive pathways and the subsequent mechanisms of charge transport within these pathways. Regarding the first dimension, research origins can be traced to the phenomenon of conductive percolation (Figure 1a), which is theoretically grounded in percolation theory [51,52]. Once a conductive circuit is established, two primary theoretical frameworks explain the occurrence of conductivity. The first is the conductive pathway theory (Figure 1(bI)), though it is notable that conductivity phenomena can manifest even when conductive particles have not yet formed continuous chains and interparticle distances remain relatively large [53]. The second framework comprises micro-tunnel current theory (Figure 1(bII)) and the field emission mechanism (Figure 1(bIII)), which have gained broader acceptance in the field [54]. Within the tunnel current theory, particularly relevant to binary conductive composites, when the concentration of the highly conductive phase approaches the percolation threshold, electron tunneling conduction emerges as a dominant mechanism influencing the material’s electrical behavior. This theory posits that charge transport occurs not through direct physical contact between conductive particles but via quantum tunneling of electrons across the nanoscale gaps separating them. Medalla [55] states that under low-temperature conditions, the electronic tunneling current density satisfies the following Equation (1):

(1) $$j(\varepsilon )=j_{0}\exp\left[-\frac{\pi \chi \omega }{2}{\left(\frac{\left|\varepsilon \right|}{\varepsilon_{0}}-1\right)}^{2}\right]|\varepsilon |<\varepsilon_{0}$$

Among them, *ε* is the electric field strength between the conductive particles, *j*(*ε*) is the electron tunneling current density, *j*_0_ is the equivalent conductivity of the gap, *χ* is the Barrier correlation coefficient, *ω* is the gap width, *ε*_0_ is the characteristic electric field strength.

Sheng [56] studied the relationship between tunnel current theory and temperature, suggesting that under low temperature and low pressure conditions, the relationship between tunneling conductivity and temperature can be expressed by the following Formula (2):

(2) $$\sigma =\sigma_{0}exp\left[-T_{1}/\left(T+T_{0}\right)\right]$$

*σ* and *σ*_0_ are the resistivity of the composite material and the conductivity of the high-conductivity component, respectively. *T*_1_ and *T*_0_ are parameters related to temperature. When the concentration of conductive fillers is increased, both the material resistivity *σ* and the parameter *T*_1_ decrease; it is also found that the sensitivity of resistivity to temperature decreases.

Van Beek [57] also used the electronic tunneling effect to explain the conductive behaviors of particle composite conductive materials. He believed that this type of conductive behavior is generated by the emission of the internal electric field of the conductive particles. Although insulators exist between the conductive particles, when the distance between conductive particles is less than 10 nm, the strong electric field formed between the particles can produce an emission electric field, thus generating current. This theory is also known as the field emission theory. Its main equation is represented by the Formula (3):

(3) $$J=AE^{m}exp\left(-\frac{B}{E}\right)$$

*J* is the current density, *E* is the electric field strength, *A* is the tunneling frequency, *m* and *B* are the characteristic constants of the composite material. The value of *m* generally ranges from 1 to 3. Temperature and the concentration of conductive fillers have a relatively small influence on the field emission theory. Its application range is broader than that of the percolation theory and the effective medium theory and it can reasonably explain the non-Ohmic characteristics of many composite materials.

The theory of the field emission effect is like that of the tunnel current theory, occurring between conductive particles that are relatively close to each other. Electrons are ionized under the influence of an external electric field. The ionized electrons cross the potential barrier of the polymer to move and generate current. The field emission effect is a special case of the tunnel current theory. The field emission effect only occurs when conductive polymer materials are influenced by external factors, such as local heating or excessively high local electric fields [58]. The above theory sequentially reveals the conductive mechanisms when conductive fillers are in full contact, close contact, and at a certain distance, providing a comprehensive explanation of the physical structural basis of the conductive permeation phenomenon, as well as explaining the reasons for the path construction of current polymer-based conductive materials under different conditions. This theory provides theoretical guidance for the microstructural optimization of polymer composite conductive materials.

**Figure 2:**
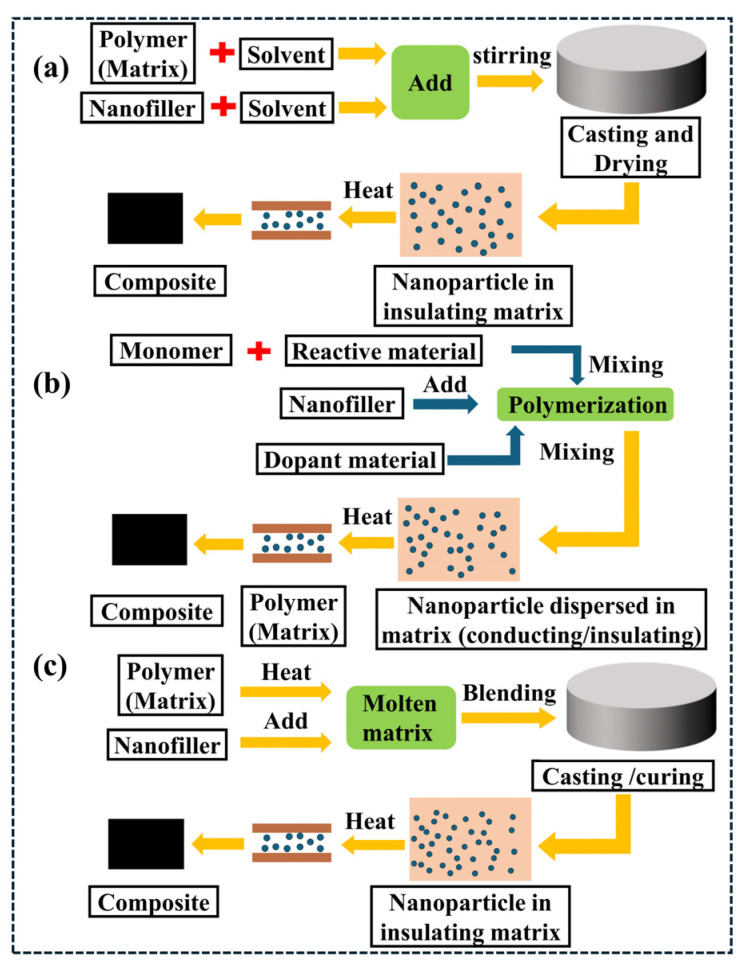
(**a**) Preparation of conductive insulating polymers by in situ polymerization, (**b**) method for preparing exogenous polymers by the solution mixing method, (**c**) method for preparing polymer composites by the melt mixing method [75].

The electrical conductivity of conductive polymer composites (CPCs) is primarily governed by the synergistic interactions among the polymer matrix, conductive fillers, and processing technologies. Key properties of the polymer matrix, including surface tension, crystallinity, and relative molecular mass, collectively influence the electrical conductivity of the composite system [59,60,61,62,63]. Meanwhile, the matrix’s polarity, crystallinity, and viscosity impact filler dispersion, whereas the type, morphology, and surface functionalization of conductive fillers dictate the percolation threshold and connectivity of the conductive network. For a fixed filler content, higher crystallinity in the composite system increases the effective filler concentration within the amorphous regions, thereby enhancing the material’s electrical conductivity [64,65]. Once the conductive filler content surpasses the percolation threshold, filler particles form a continuous three-dimensional conductive network within the matrix, establishing effective electron conduction paths and conferring favorable electrical conductivity to the material [66,67,68,69]. Furthermore, during composite fabrication, process parameters, including temperature, pressure, processing time and mixing speed, significantly influence the dispersion state and distribution uniformity of conductive fillers within the polymer matrix, directly impacting the material’s final electrical conductivity [70,71]. Commonly employed preparation methods for composite conductive polymer materials include in situ polymerization [72,73], solution blending [74], and melt blending [75] (Figure 2).

For instance, Wang [73] fabricated lightweight carbon nanotube/polyimide (CNT/PI) foam by introducing carbon nanotubes (CNTs) into soluble polyamic acid (PAA), followed by in situ polymerization of PAA into polyimide (PI). The resulting CNT/PI foam exhibited a low density of 32.1 mg/cm^3^, alongside a high electromagnetic interference shielding effectiveness (EMI SE) of approximately 41.1 dB and an absorption coefficient reaching 82.3%. Chiu [74] successfully synthesized ternary nanocomposites by incorporating graphene nanoplatelets (GNPs) and carbon nanotubes (CNTs) into a miscible blend of polyvinylidene fluoride (PVDF) and polyvinyl acetate (PVAc), notably enhancing the material’s electrical conductivity. Rafeie [76] investigated the interrelationships among rheology, morphology, and conductivity in the polyvinylidene fluoride/polyethylene/graphene nanoplatelet (PVDF/PE/GNP) ternary system. A series of blends was fabricated via a two-step melt blending method, where 1.5 wt% GNP enabled the formation of a conductive network within the PVDF matrix, yielding a conductivity of approximately 10^−2^ S/cm. Similarly, Chen [77] utilized melt blending to combine polystyrene (PS), polymethyl methacrylate (PMMA) and multi-walled carbon nanotubes (MWCNTs), fabricating conductive composites through controlled processing temperatures and speeds. Subsequent supercritical CO_2_ foaming treatment produced materials characterized by low density (0.4 g/cm^3^), high electrical conductivity (1.64 S/m), and efficient electromagnetic interference shielding performance (23.08 dB).

## 3. Conductive Polymer Microcellular Foaming Mechanism

### 3.1. Supercritical Carbon Dioxide

**Figure 3:**
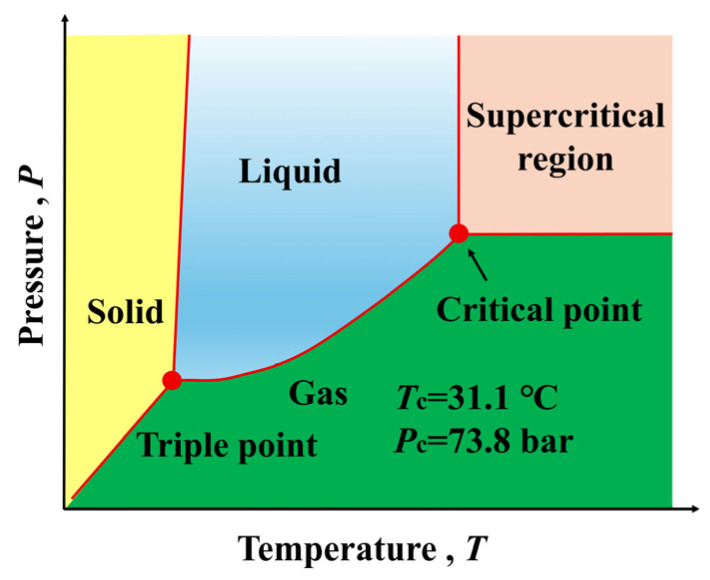
CO_2_ phase diagram. Reproduced with permission from Ref. [24]. Copyright 2023 Springer Nature.

Pure substances exhibit three different states: gas, liquid, and solid, depending on changes in temperature and pressure. When the temperature and pressure reach the critical point, the liquid–gas interface disappears and, at this point, the physical properties of the fluid, such as density, viscosity, and solubility, change dramatically. Liquids that are above the critical point are referred to as supercritical fluids, which have characteristics such as low viscosity and high diffusion coefficient, and these properties are sensitive to changes in temperature and pressure [78]. When CO_2_ is above 31.1 °C and 7.38 MPa (Figure 3) [79], it is in a supercritical state, making the supercritical conditions of CO_2_ easier to achieve. Supercritical carbon dioxide (sc-CO_2_) serves as a green solvent, with advantages such as chemical inertness, no residues, and recyclability, preventing material degradation. It is widely applied in polymer material processing and modification, as well as in the preparation and foaming of micro–nano materials [23,80,81,82].

### 3.2. Principle of the Foaming Process

**Figure 4:**
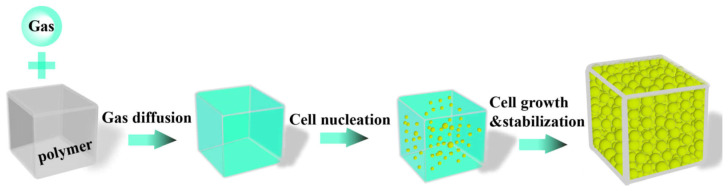
Schematic diagram of supercritical fluid foaming. Reproduced with permission from Ref. [24]. Copyright 2023 Springer Nature.

The conductive polymer microcellular foaming process follows the classical gas-induced phase separation mechanism, with its core stages including the formation of a polymer/gas homogeneous system, cell nucleation, cell growth, and a stable basic phase (Figure 4). This process begins with the thorough mixing of the polymer matrix and supercritical fluid, under specific temperature (*T*) and pressure (*P*) conditions, where gas molecules penetrate into the gaps between polymer chains through diffusion until a thermodynamic equilibrium state is reached, forming a homogeneous saturated system [83]. In this stage, the plasticizing effect of supercritical fluids can significantly lower the glass transition temperature (*T*_g_) of polymers, enhancing the mobility of molecular chain segments, thereby reducing the free energy barrier required for cell nucleation [84]. The essence of cell nucleation is a phase separation process driven by the thermodynamic instability of the system [85]. The core driving force of the cell growth stage originates from the diffusion behavior of gas in a supersaturated system [86]. When the gas feeding rate falls below the demand for pore expansion or the viscoelastic properties of the polymer matrix reach a critical threshold, pore growth stagnates, ultimately yielding a cell architecture characterized by a well-defined size distribution and structural density [87].

#### 3.2.1. Formation of a Polymer/Gas Homogeneous System

The formation of a uniform polymer/gas system during microcellular foaming lays the thermodynamic foundation for subsequent cell nucleation and growth. The microcellular process occurs through the dissolution and diffusion of physical foaming agents (such as supercritical CO_2_ or N_2_), ultimately achieving a thermodynamically metastable state of saturation. The progress of this system is controlled by a dissolution diffusion coupling mechanism, which coordinates the interaction between the solubility of foaming agents in polymers and their subsequent diffusion behavior [88]. The solubility is jointly regulated by the type of foaming agent, the polymer matrix, and the temperature/pressure conditions. At a given temperature, the local concentration *C* of the gas dissolved in the polymer is related to the pressure [89], as described in Equation (4):

(4) $$C=S\left(C\right)P$$

Herein, *C* is the solubility of the gas in the polymer melt, *S*(*C*) is the solubility coefficient, *P* is the saturated pressure.

The solubility of the gas is related to temperature [90]. The formula is as follows (5):

(5) $$S=S_{0}e^{\frac{-\Delta H_{s}}{RT}}$$

where *S*_0_ is the exponential factor, Δ*H*_s_ is the enthalpy of adsorption, *T* is the temperature, *R* is the gas constant. Since Δ*H*_s_ is negative, the solubility of the gas decreases with increasing temperature [91].

According to Henry’s law, when pressure and temperature are constant, the solubility of gases and their solubility limits in polymers are both fixed values. To ensure the foaming process forms a homogeneous system, the pressure applied is usually much higher than the gas saturation pressure, and so understanding gas solubility is crucial [92,93]. Apart from solubility, the gas diffusion rate affects the formation time of the homogeneous system and the growth rate of cells. This rate is mainly regulated by temperature and is directly proportional to it. The diffusion coefficient (*D*) of gas in the polymer can be calculated using the relevant Formula (6) [94]:

(6) $$D=D_{0}\exp\left(-\frac{E_{a}}{R_{g}T}\right)$$

where *E*_a_ is the diffusion activation energy, *R*_g_ is the gas constant, and *D*_0_ is the pre-exponential factor of the diffusion coefficient.

Sun [95] studied the effects of CO_2_ solubility and diffusion rates in polypropylene (PP) and composite PP materials containing different contents of carbon nanofibers (CNF). The study found that as the CNF content increased, the solubility and diffusion rates of CO_2_ in the polymer also increased. This is because CNF can absorb a small amount of CO_2_ and can cause changes in the free volume of the polymer matrix. Areerat [96] investigated the diffusion rates and solubility of sc-CO_2_ in molten states of low-density polyethylene (LDPE), high-density polyethylene (HDPE), polypropylene (PP), ethylene-vinyl acetate (EEA), and polystyrene (PS) using the magnetic suspension balance method (MSB). The results showed that with an increase in temperature, the solubility of CO_2_ decreased, but with an increase in pressure, the solubility of CO_2_ increased.

#### 3.2.2. Cell Nucleation

Cell nucleation is the process by which gas molecules aggregate from a supersaturated solution to form initial bubbles, driven mainly by concentration gradients, pressure gradients, or temperature gradients [25,97]. By reducing gas solubility through depressurization or heating, solution supersaturation can be achieved. When gas molecular clusters exceed the critical size, the system overcomes the energy barrier to nucleate, and the degree of supersaturation is negatively correlated with the nucleation activation energy. The classical nucleation theory was refined by Milton and Blander, later expanded to the field of microcellular plastics by Colton and Suh, establishing a bubble nucleation model applicable to foam plastics, providing a theoretical framework for understanding the initial stage of conductive polymer microcellular foaming [98].

(1) Homogeneous Nucleation

Homogeneous nucleation refers to the process of spontaneously forming cells in a homogeneous system. It needs to overcome the maximum free energy barrier and requires the system to have a high supersaturation. This process is jointly determined by the decrease in volume free energy (driven by gas precipitation) and the increase in surface free energy (resistance from new interface formation). The Gibbs free energy change before and after the formation of the nucleus follows the classical thermodynamic Formula (7).

(7) $$\Delta G=-\frac{4}{3}\pi r^{3}\Delta P+4\pi r^{2}\sigma$$

Here, *σ* represents the surface tension at the interface between the polymer and the cell, *r* is the radius of the bubble nucleus, and Δ*P* is the pressure difference inside and outside the cell. The first term on the right side of the formula is volumetric free energy, and the second term is the interfacial energy that must be overcome for the cell to grow.

When the size of the bubble nucleus is larger than the critical nucleation radius r*, the bubble nucleus can continue to grow and form a cell. The critical radius r* is determined by the ratio of the interfacial tension *σ* to the pressure difference Δ*P*, as shown in Equation (8). The Gibbs free energy of homogeneous nucleation, ΔG*_hom_ is expressed by Equation (9):

(8) $$r^{*}=\frac{2\sigma }{\Delta P}$$

(9) $$\Delta G_{\mathrm{hom}}^{*}=\frac{16\pi \sigma^{3}}{3\Delta P^{2}}$$

The classical homogeneous nucleation theory is deduced from the theoretical derivation of pure vapor-forming liquid droplets [99]. Colton et al. [100] calculated the homogeneous nucleation rate of gaseous polymers (*N*_hom_), as shown in Equation (10):

(10) $$N_{\mathrm{hom}}=C_{0}f_{0}exp\left(-\frac{\Delta G_{\mathrm{hom}}^{*}}{kT}\right)$$

Among them, *C*_0_ is the gas concentration, *f*_0_ is the vibration frequency of gas molecules, *K* is the Boltzmann constant. From Equations (11) and (12), it can be found that a higher gas adsorption amount, a larger pressure drop, and a higher foaming temperature can achieve a higher cell nucleation rate, thereby enhancing cell nucleation. Kumar [101] found in the study of poly(vinyl chloride) (PVC) that as the temperature increases, the number of nuclei increases. In contrast, Goel [102] found that in the PMMA-CO_2_ system, the number of nuclei formed decreases with increasing temperature. Meanwhile, Baldwin [103] found that when the temperature is below 100 °C, the number of nuclei in amorphous poly (ethylene terephthalate) (APET) and crystalline poly (ethylene terephthalate) (CPET) increases with the rise in foaming temperature. However, when the foaming temperature exceeds 100 °C, the number of nuclei decreases as the temperature rises. In addition, in semicrystalline PET and CPET, the number of nuclei changes slightly with temperature. Based on the above phenomena, researchers believe that gas nuclei are mainly formed during the heating stage or the pressure relief process.

(2) Heterogeneous nucleation

**Figure 5:**
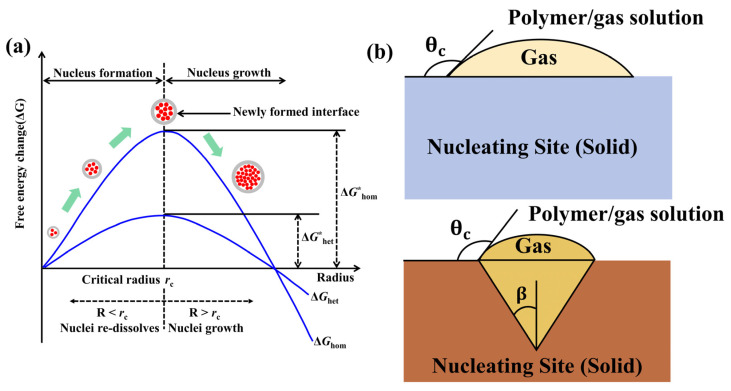
(**a**) Schematic diagram of the free energy barrier of homogeneous nucleation and heterogeneous nucleation. (**b**) Schematic diagram of out-of-phase nucleation at smooth planes and holes. Reproduced with permission from Ref. [24]. Copyright 2023 Springer Nature.

Different from homogeneous nucleation, heterogeneous nucleation generates nucleation sites preferentially at phase interfaces, those formed between nucleating agents, additives, polymer crystals, initiator residues and the polymer matrix [104,105,106], where the free energy barrier of such interfaces is lower than that required for homogeneous nucleation (Figure 5a).

Due to the complexity of heterogeneous nucleation theory, its basic nucleation mechanism has not been thoroughly studied. However, it has been proven that adding additives or nucleating agents can promote heterogeneous nucleation and simultaneously increase the cell density of foamed samples [107].

The heterogeneous nucleation rate (*N*_het_) is shown in Equation (11):

(11) $$N_{\mathrm{het}}=C_{1}f_{1}exp\left(-\frac{\Delta G_{\mathrm{het}}^{*}}{kT}\right)$$

In the formula, *C*_1_ is the content of gas molecules in unit volume of melting, *f*_1_ is the number of effective collisions per unit time and unit volume between gas molecules and gas nuclei. *K* is the Boltzmann constant, *T* is the absolute temperature, ΔG*_het_ is the Gibbs free energy barrier for heterogeneous nucleation.

When many second-phase particles exist in the polymer/gas system, heterogeneous nucleation dominates the cell nucleation mode. The interface between the second-phase particles and the polymer can significantly reduce the nucleation resistance. Thus, compared with homogeneous nucleation, heterogeneous nucleation has a lower nucleation energy barrier. By modifying the critical nucleation energy barrier of homogeneous nucleation with the heterogeneous factor, the critical nucleation energy barrier of heterogeneous nucleation ΔG*_het_ can be obtained, as shown in Equation (12):

(12) $$\Delta G_{\mathrm{het}}^{*}=\Delta G_{\mathrm{hom}}^{*}F\left(\theta \right)$$

where *θ* is the wetting angle between the cell and the heterogeneous interface. Blander [108] derived the formula for calculating the heterogeneous factor *F*(*θ*), as shown in Equation (13):

(13) $$F\left(\theta \right)=\frac{\left(2+\cos\theta \right){\left(1-\cos\theta \right)}^{2}}{4}$$

The geometric shapes of the nucleation sites vary due to factors such as the nucleating agents themselves, additives, or impurities. If not all of the added nucleating agents have smooth surfaces, gas nuclei may form in the conical cavity (Figure 5b). The half-cone angles *β* at different nucleation positions are distributed between 0~90°.

In this case, *F*(*θ*_c_, *β*) is the reduced energy barrier. *F*(*θ*_c_, *β*) can be expressed as Equation (14):

(14) $$F\left(\theta_{c},\beta \right)=\frac{1}{4}\left[2-2\sin\left(\theta_{c}-\beta \right)+\frac{\cos\theta_{c}\cos^{2}\left(\theta_{c}-\beta \right)}{\sin\beta }\right]$$

In the polymer foaming process, homogeneous nucleation and heterogeneous nucleation coexist simultaneously, which can be expressed by the following Equation (15):

(15) $$N=N_{\mathrm{hom}}+N_{\mathrm{het}}$$

In the heterogeneous nucleation process, in addition to the factors influencing homogeneous nucleation, the type, shape, number, and density of the second-phase particles also have a remarkable impact on the nucleation process. Moreover, it is noteworthy that the crystalline phase inherent in the polymer itself may also serve as a heterogeneous nucleating agent [109,110].

#### 3.2.3. Cell Growth

**Figure 6:**
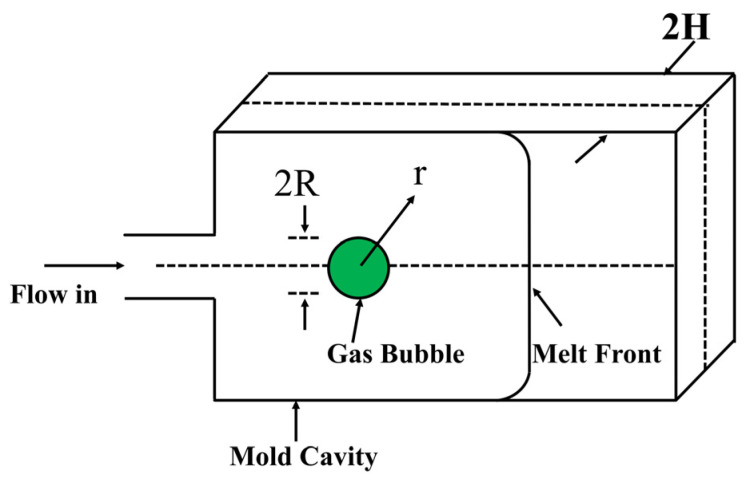
“Sea-island” model. Reproduced with permission from Ref. [24]. Copyright 2023 Springer Nature.

Cell growth is a mass transfer process dominated by gas diffusion and is affected by the coupling effects of multiple factors, such as melt pressure, temperature, surface tension, and gas content [111,112]. Owing to the inherent complexity of polymer melts and the intricate mass transfer mechanisms between the gas and melt phases, it remains challenging to precisely characterize the dynamics of cell growth. To overcome these difficulties, several theoretical models have been proposed, such as the “Sea-island” model developed by DeWitt and Leonov [113] and Amon’s cell model [114]. The Sea-island model describes the growth behavior of a single cell in an infinite melt (Figure 6). However, the Sea-island model only targets the growth of a single cell in an infinitely large melt, while in cell nucleation, cases that conform to the Sea-island growth model are rare. In fact, during cell nucleation, the dissolved gas in the polymer is in a supersaturated state. Thermodynamic instability leads to the instantaneous generation of many cells, and the small spacing between cells differs greatly from the condition in the Sea-island model, where a single cell is infinitely melted. Therefore, the Sea-island model is not suitable for simulating actual cell growth.

**Figure 7:**
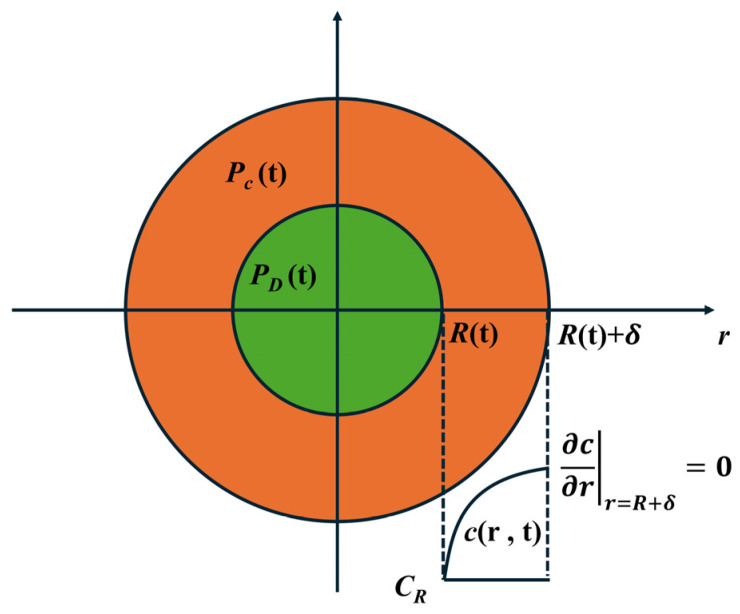
Cell model of bubble growth. Reproduced with permission from Ref. [24]. Copyright 2023 Springer Nature.

The cell model describes the mass and momentum transfer of a single cell and within the limited volume shell surrounding it. This model confines the gas that can diffuse into the cell to the cell wall shell. It is assumed that the cell is surrounded by a concentric, constant mass melt spherical shell, in which a large amount of gas is dissolved. During the cell expansion process, the diffusion of the gas only occurs between the gas dissolved in the melt of the cell wall shell and the cell (Figure 7). The cell model addresses the shortcomings of previous models. By regarding the entire foamed material as being composed of a large number of spherical cells, it more realistically reflects the interrelationships during the simultaneous expansion of adjacent bubbles in the melt and effectively resolves the interrelationships in the expansion process of adjacent bubbles.

In simulation studies, the most commonly used physical model is the cell model. During the cell growth process, the gas in the surrounding melt continuously diffuses into the cell, the gas concentration in the melt continuously decreases, and thus a concentration boundary layer is formed. Outside the concentration boundary layer, the gas concentration remains unchanged.

According to the cell model, under isothermal conditions, the kinetics of foam cell growth can be described by coupled mass and momentum conservation equations. The mass conservation of gas inside the cell can be expressed as (16):

(16) $$\frac{d}{dt}(\frac{4\pi }{3}\frac{P_{D}R^{3}}{R_{g}T})=4\pi R^{2}D\frac{\partial c}{\partial r}{|}_{r=R}$$

The mass conservation of gas within the melt can be expressed as (17):

(17) $$\frac{\partial c}{\partial t}+\frac{\dot{R}R^{2}}{r^{2}}\frac{\partial c}{\partial r}=\frac{D}{r^{2}}\frac{\partial }{\partial r}\left(r^{2}\frac{\partial c}{\partial r}\right)$$

Momentum conservation Equation (18):

(18) $$P_{D}-P_{C}-\frac{2\gamma }{R}+2\int_{R}^{\infty }\frac{\sigma_{rr}-\sigma_{\theta \theta }}{r}dr=\frac{4\eta }{R}\left(\frac{dR}{dt}\right)$$

Here, *R* represents the cell radius, $\dot{R}$ is the cell growth rate, *D* is the diffusion coefficient, *c* is the gas concentration, *η* is the melt viscosity, *R*_g_ is the gas constant, *σ* is the viscoelastic stress tensor.

Comparing the two models, the cell model can more accurately represent the cell growth process; thus, it is widely used for simulating the cell growth process. Some other scholars further considered various influencing factors and modified and improved the equations describing cell growth. For example, they considered viscoelastic effects, non-isothermal conditions, non-isobaric conditions, and gas loss [115,116,117]. Other scholars combined the nucleation process and bubble growth process to identify the key factors affecting cell structural parameters. Specifically, the factors affecting cell growth mainly include the physical property parameters of the foaming system (such as gas type, solubility, gas diffusion coefficient, melt viscosity, gas—polymer interfacial tension, etc.) and processing parameters (such as temperature, pressure, shear rate, additives, etc.) [118,119].

Cell stability refers to the process of maintaining the cell structure without collapse or coalescence during the foaming process. Curing is to convert liquid or molten foam into a solid state through physical or chemical methods, thereby fixing the cell morphology. Naguib et al. [120] believed that there is a curing temperature that maximizes the foaming ratio. When the curing temperature is too high, the gas diffusion rate is high, gas loss is significant, and coalescence may occur between cells, resulting in a reduced foaming ratio. When the curing temperature is too low, the crystallization rate of the sample is too fast, bubbles are fixed before they can grow, and so a high foaming ratio cannot be achieved either. Therefore, choosing an appropriate curing temperature during curing is particularly important.

## 4. Foam Manufacturing Techniques

Conductive polymer microcellular foaming technology mainly includes three molding processes: batch foaming, extrusion foaming, and injection foaming. The following section briefly discusses these three widely used foam processing methods. Each method has its unique technical characteristics and procedures. It should be noted that although the processing methods differ, the basic foaming principles remain the same regardless of which of the above methods is adopted [121,122].

### 4.1. Batch Foaming

The batch foaming process is conducted in a temperature-controlled pressure vessel. Initially, the polymer sample is sealed in a high-pressure autoclave and supercritical CO_2_ or N_2_ is introduced. By synergistically controlling temperature, pressure and time parameters, the blowing agent attains a solubility equilibrium within the polymer matrix, thereby forming a homogeneous saturated system. Subsequently, this thermodynamic equilibrium is broken via rapid depressurization or stepwise heating: a sudden decrease in the blowing agent’s solubility triggers cell nucleation and then, gas diffusion takes the lead in cell growth. Eventually, the cell structure is regulated by the coupling of multiple parameters such as saturation pressure, depressurization rate, and cooling rate, resulting in characteristic structures like cell size distribution and the ratio of open to closed cells [123,124,125].

**Figure 8:**
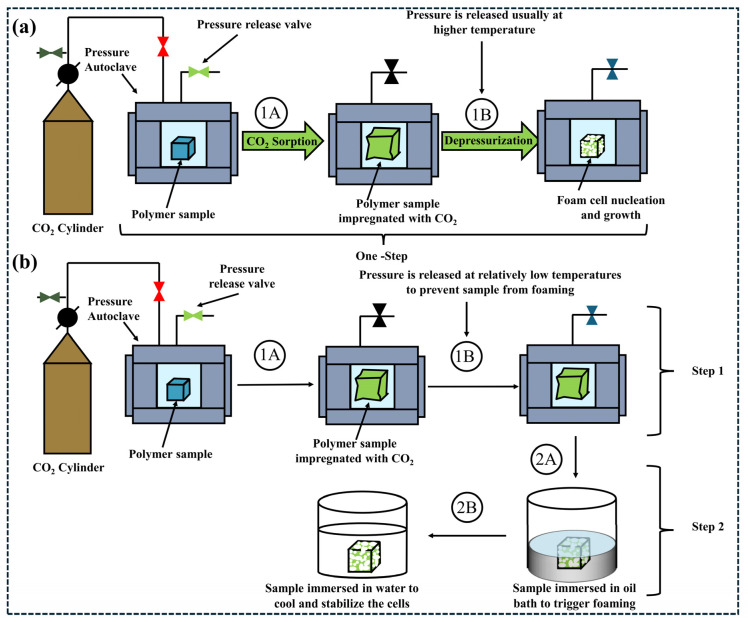
(**a**) Pressure-induced batch foaming process (Δ*P*/Δ*t*), (**b**) temperature-induced batch foaming process (Δ*T*/Δ*t*) (adapted from [27]).

Pressure-induced batch foaming process realizes gas saturation at a temperature higher than the polymer’s *T*_g_ (glass transition temperature). After depressurization, rapid supersaturation leads to bubble formation, which is then quenched to solidify the structure (Figure 8a). In contrast, thermally induced foaming involves transferring the sample to temperature-induced batch foaming (above *T*_g_) after low-temperature saturation to induce supersaturation. Both processes require quenching to room temperature to stabilize the structure. However, pressure-induced batch has emerged as the dominant technique for batch foaming due to its convenient operation, short processing time, and controllable cell structure (Figure 8b) [26].

Jun [126] prepared stretchable MWCNT/TPU conductive composite foams by using sc-CO_2_ static batch foaming technology. He systematically investigated the modulation mechanism of cell structure on electromechanical properties. By adjusting the saturation temperature, gradient control of cell density was achieved, and the change in port density led to a non-monotonic response in electrical conductivity. Salari [127] transformed MWCNTs into GNRs through the chemical unzipping method. Combined with solvent blending and batch foaming technology, PVDF-based nanocomposites were prepared. By regulating the microcellular structure to promote the oriented arrangement and connection of nano-fillers, the electrical conductivity was further improved. The combination of foaming and optimization of nano-filler morphology can reduce the filler dosage while enhancing the electromagnetic shielding effectiveness.

While batch foaming offers the best controllability over cell structure, it is not well-suited for high-throughput production and large-scale manufacturing. The method excels in laboratory settings where precise morphology control is crucial but is limited by its relatively low production capacity. Supercritical fluid foaming, especially using CO_2_, provides better scalability and the ability to control bubble size and uniformity more easily than chemical foaming, making it a more industrially viable option for larger productions. However, the process complexity and equipment cost can still pose challenges, especially when large batches are involved [23].

### 4.2. Extrusion Foaming

**Figure 9:**
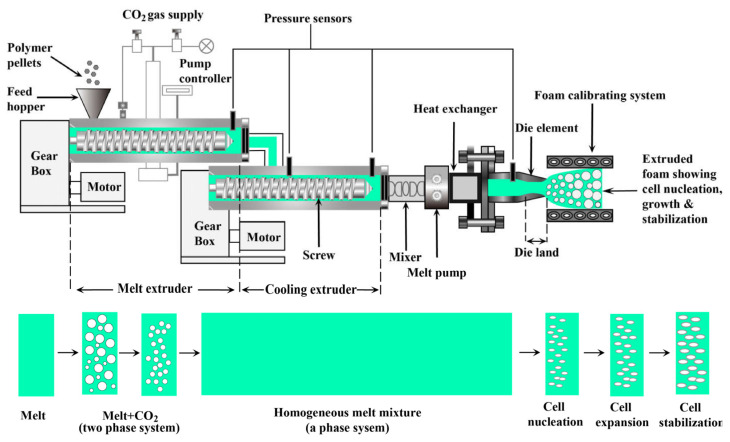
Schematic representation of foam extrusion on a tandem line. Reproduced with permission from Ref. [24]. Copyright 2023 Springer Nature.

The extrusion foaming process realizes the continuous mixing of polymer melt and sc-CO_2_ in a twin-screw extruder. After the polymer raw material enters the extruder barrel via the feeding system, sc-CO_2_ is injected at the high-pressure section and fully mixed with the melt. A homogeneous saturated system is formed through the control of the multi-stage shear field and temperature gradient (Figure 9). When the polymer/gas mixture is extruded through the die head, the sudden pressure drops to the atmospheric pressure triggers dual mechanisms: gas expansion induces cell nucleation and growth and, at the same time, conductive fillers rearrange and are oriented along the bubble walls under the action of interfacial tension to form a three-dimensional conductive network. Subsequently, the gradient cooling system rapidly solidifies the cell structure to suppress gas escape and finally, lightweight functional materials with uniform cell distribution and stable electrical conductivity are prepared [128].

Jeon [129] systematically investigated the dynamic regulation mechanism of the electrical conductivity of carbon fiber/polycarbonate (CF/PC) conductive composites via a microcellular foaming process. This study revealed the nonlinear influence laws of cell size (1–100 μm) and foaming ratio (5–50%) on the volume resistivity of the material by comparing batch foaming and continuous foaming (injection molding) processes, providing a process paradigm for the precise regulation of functional conductive foams. Ghanemi [130] prepared LDPE/EVA blends containing carbon nanotubes (CNTs) through the twin-screw extrusion process, using the semiconductive foam of low-density polyethylene/ethylene–vinyl acetate copolymer (LDPE/EVA) as the base. The effects of CNTs’ content and their distribution state in the blend on the physical and structural properties of LDPE/EVA/CNTs foamed materials were investigated. The results show that as the CNTs’ content increases, the porosity and cell density of LDPE/EVA/CNTs foamed materials increase.

Extrusion foaming excels in continuous production, making it more suitable for industrial-scale manufacturing, but it sacrifices fine control over bubble size and uniformity compared to batch foaming. The shear forces involved in extrusion can lead to variations in the foam structure, and the process requires optimal management of shear rates, gas content, and temperature gradients to ensure consistent foam quality. This limits its ability to achieve the same level of precise control that batch foaming can provide [122].

### 4.3. Injection Foaming

**Figure 10:**
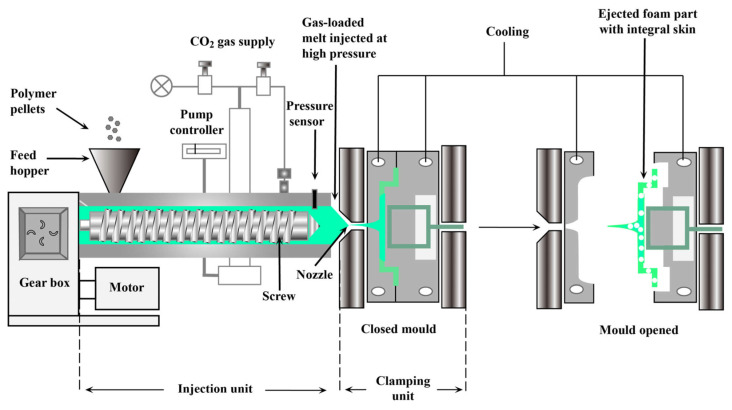
Schematic representation of foam injection. Reproduced with permission from Ref. [24]. Copyright 2023 Springer Nature.

The injection molding foaming technology of conducting polymers realizes the synergistic preparation of material lightweighting and functionalization by integrating conductive fillers and foaming processes. This process uses sc-CO_2_ as a physical foaming agent, uniformly dispersing the gas in the conducting polymer matrix in the screw melting zone to form a gas–solid two-phase system (Figure 10). During the injection stage, cell coalescence is suppressed through precise pressure control. Rapid die cooling promotes the formation of a dense conductive network on the surface layer, while a gradient cell structure develops in the core layer. The oriented distribution of conductive fillers on the cell walls constructs three-dimensional conductive pathways, endowing the material with electromagnetic interference shielding effectiveness (EMI SE) while reducing its density, balancing mechanical strength and functional characteristics through precise control of process parameters [131,132,133].

Ameli [134] conducted foam injection molding experiments to determine the relationship between processing, microstructure, and electrical conductivity of polypropylene/multi-walled carbon nanotube (PP/MWCNTs) nanocomposites. They studied the effects of injection flow rate, gas content, melt temperature, void ratio, and cavity position on microstructure and electrical conductivity. Under optimal processing conditions, foam materials with cellular skin and core regions were obtained, whose electrical conductivity was six orders of magnitude higher than that of the solid materials. Electrical conductivity increased positively with the increase in injection flow rate and reached its maximum under optimal values of gas content (0.3%), melt temperature (200 °C), and void ratio (30%). Li [135] investigated the injection foaming process of in situ fibrillation-reinforced polypropylene composites. With polypropylene (PP) as the continuous phase, polytetrafluoroethylene (PTFE) as the dispersed phase, multi-walled carbon nanotubes (MWCNTs) as the conductive filler, and polypropylene grafted with maleic anhydride (PP-g-MA) as the compatibilizer, MWCNTs/PP-g-MA master batches were prepared by the solution blending method. Subsequently, light-conductive PP/PTFE/MWCNTs composite foamed materials were prepared through extrusion pelletizing and supercritical nitrogen (sc-N_2_) injection foaming processes. The composite foamed materials were systematically analyzed in terms of rheological properties, micro-morphology, foaming behavior, mechanical properties. The results showed that the in situ fibrillation of PTFE could significantly improve melted strength and viscoelasticity, thereby enhancing the foaming performance.

Injection foaming is suitable for complex part geometries and offers a unique advantage in achieving a skin-core structure with distinct properties in different layers. However, it is technically demanding and requires strict control over processing conditions such as injection flow rate, gas content, and melt temperature to prevent cell coalescence and ensure uniform foam quality. Despite its flexibility, injection foaming is not as widely used as extrusion foaming in large-scale production due to the difficulty in optimizing the foam structure across various part geometries [24].

Despite significant advancements in conductive polymer microcellular foaming, several technical bottlenecks remain: the difficulty of simultaneously achieving ultrafine cells and low density. Achieving both high foam density and small cell size remains a challenge, as these factors are often inversely related. In addition, insufficient melt strength in many polymers results in poor cell wall stability during foaming, leading to cell collapse and coalescence. Poor foamability of semicrystalline systems: Semicrystalline polymers have lower gas solubility and experience hindered bubble growth, making foamability more difficult and leading to non-uniform structures. Limited understanding of the coupling between rheology, thermal history, and gas transport: the interplay of these factors is still not well understood, which limits the ability to fully optimize foaming processes. Gap between laboratory-scale morphology optimization and industrial-scale manufacturing: although batch foaming provides excellent morphology control, it is not suitable for industrial-scale production due to its low throughput. Extrusion and injection molding foaming are more scalable but face challenges in achieving uniform foam structures at large scales.

## 5. Applications of Microcellular Foaming for Conductive Polymers

### 5.1. Electromagnetic Interference Shielding

Compared with traditional solid structures, the cell structure introduced by various foaming methods is considered one of the most effective means to improve electromagnetic interference shielding [136,137]. This method can not only effectively regulate the distribution of fillers but also optimize the microstructure of conductive polymer composites (CPCs), thereby significantly enhancing their electromagnetic interference shielding (EMI Shielding) performance [138]. The cell structure of CPCs not only strengthens the conductive network by introducing many cells but also improves the electromagnetic interference shielding effectiveness (EMI SE) by forming multiple interfaces. Typically, such microcellular structures are obtained by introducing gas or other media into the polymer matrix to form a mixed system and then removing the media through subsequent treatment [139,140]. Currently, most research primarily focuses on regulating the cell size of the foam or the material thickness [141,142]. In terms of regulating shielding performance, existing studies mostly achieve this by introducing a gradient distribution of fillers or adjusting the processing parameters during preparation [143]. However, the methods generally have certain application limitations, mainly reflected in the fact that the electromagnetic shielding performance of the prepared samples is usually static and fixed, making it difficult to respond to changes in electromagnetic waves in different environments. In contrast, dynamically adjusting EMI shielding performance in response to external stimuli from composite materials themselves shows greater potential in practical applications. Therefore, developing responsive composite materials that can intelligently regulate EMI shielding performance under external stimuli such as temperature, stress, pH, electric fields, or magnetic fields has become an important development direction and urgent need in this field [144].

**Figure 11:**
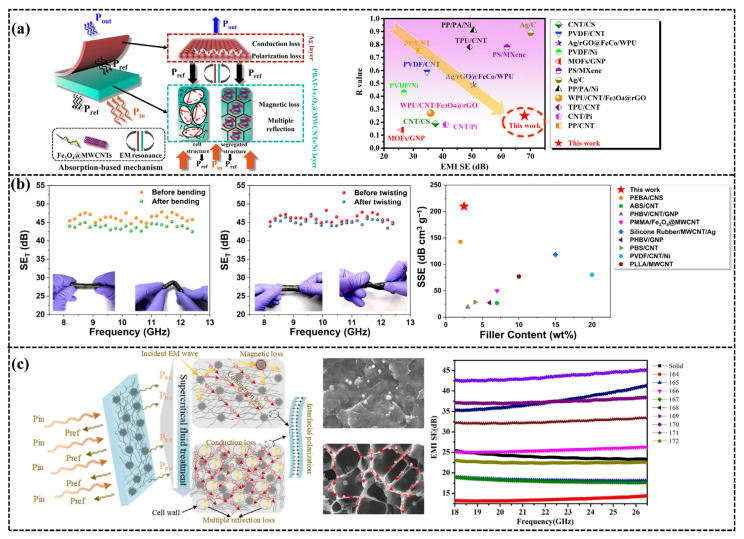
(**a**) Scheme of EM wave dissipation mechanism within the composite foams. Comparison of EMI SE and R value of EMI shielding materials [148]. (**b**) EMI shielding effectiveness before and after bending and twisting 1000 times. EMI shielding performance of different composite foam systems fabricated using sc-CO_2_ foaming [150]. (**c**) Illustrates the schematic diagram of the microwave shielding mechanism in solid and foamed PVDF/CNTs/urchin-like composites, as well as the electromagnetic interference (EMI) shielding effectiveness of PVDF/10 wt% CNTs/10 wt% Ni foams within the frequency range of 18.0–26.5 GHz [154]. Under the CC BY-NC-ND 4.0 license (https://creativecommons.org/licenses/by-nc-nd/4.0/, accessed on 9 March 2026. No modifications were made).

The large specific surface area, high porosity, and ordered cell structure of conductive polymer microcellular foaming materials play a crucial role in promoting electromagnetic wave absorption. For example, excellent impedance mismatch and an extremely high attenuation constant enable the synergistic effect between the cavity and cell wall to efficiently dissipate electromagnetic wave energy, thus developing high-efficiency microwave absorbers with low secondary reflection pollution [145]. Xie [146] prepared microcellular PSU/CNTs composite foam with an isolated structure via a combined method of solid-phase grinding and sc-CO_2_ foaming. When the CNT content is only 5.0 wt%, the foam exhibits a high electrical conductivity of 5.2 S/m, an EMI SE of 23.7 dB, and an extremely low percolation threshold of 0.06 vol%. Ma [147] systematically studied the correlation between the reflectivity (R) and thickness of microcellular conductive polymer composite (CPC) foam based on the input impedance model and successfully separated the influence of thickness on R from that of other structural variables (such as porosity VF and cell morphology). Yang [148] constructed PBAT composite foams with a layered structure through the supercritical carbon dioxide foaming and scraping techniques. This distinctive layered configuration judiciously orchestrated the merits of integrating ferroferric oxide-loaded multi-walled carbon nanotubes (Fe_3_O_4_@MWCNTs) nanoparticles, a microcellular framework, and a highly conductive silver layer, achieving maximum consumption of microwaves throughout the “absorption-reflection-reabsorption” process, which significantly declines secondary radiation pollution. The biodegradable PBAT composite foams achieved an EMI shielding effectiveness of up to 68 dB and an absorptivity of 77% and authenticated favorable stabilization after the tape adhesion experiment (Figure 11a). Jia [149] developed lightweight and compressible polymer/MXene composite foams through structural design and functionalization of MXene nanosheets; under this structure, the reflection coefficient R decreased to 0.05. Under a uniform structure, the SE reached 39.8 dB, and the absorption contribution accounted for >90%, achieving synergistic optimization of absorption-dominated electromagnetic shielding (low reflection coefficient R) and high-efficiency shielding effectiveness (SE). Lee [150] fabricated a series of nanocomposites using single-walled carbon nanotube (SWCNT) fillers. Following supercritical carbon dioxide (sc-CO_2_) foaming, a high-quality nanocomposite foam system was successfully manufactured. With the incorporation of only 2.5 wt% SWCNT, the foamed product exhibited excellent absorption-dominated electromagnetic interference (EMI) shielding performance with a specific shielding effectiveness (SSE) value of 209.8 dB cm^3^ g^−1^, which had resulted from the synergistic effect induced by the three-dimensional conductive network of SWCNT and multiple reflections inside the micropores. Deformation tests were conducted, in which the nanocomposite foam was bent or twisted 1000 times; the foam stably retained its EMI shielding capacity at more than 95% of the original performance. The nanocomposite foam was both recyclable and reformable (Figure 11b).

The main parameters of cell structures include porosity, wall thickness, cell size, and cell distribution. Changes in these parameters directly affect the variations in the foaming ratio and density of polymer foams, thereby altering the distribution of conductive fillers in the matrix [151]. Fan [152] introduced functionalized multi-walled carbon nanotubes (fMWCNTs) and liquid carboxylate nitrile butadiene rubber (CTBN) into epoxy resin (EP), prepared thermosetting composite foams via supercritical carbon dioxide (sc-CO_2_) foaming. The bidirectional stretching during cell growth promoted the oriented arrangement of fMWCNTs, forming a two-dimensional conductive network and enhancing conductivity. When the fMWCNTs content was 5.0 wt%, the foam conductivity reached 0.43 S/m, the electromagnetic interference shielding effectiveness (EMI SE) was 22.90 dB, the specific shielding effectiveness reached 37.54 dB/(g·cm^3^). This performance benefited from the synergistic effect of high conductivity and the multiple reflections of electromagnetic waves induced by the cell structure, as well as the loss of conductive fillers. Ma [153] prepared lightweight and highly efficient dual-functional insulating-type nanocomposite foam materials with microcellular structures via sc-CO_2_ foaming technology combined with hydrogen bond assembly and compression molding strategies. These materials are used for integrated infrared stealth and EMI shielding dominated by absorption. Yang [154] fabricated porous PVDF/carbon nanotubes (CNTs)/urchin-like Ni composites with different cell sizes from nanoscale to microscale through one-step supercritical carbon dioxide (CO_2_) foaming. The electrical conductivity and electromagnetic interference (EMI) shielding performance of the composites with different cell sizes were examined in detail. The results indicated that the nanoscale cell structure diminishes the EMI shielding performance of the composite, whereas the microscale cell structure with an appropriate size is beneficial for improving the EMI shielding performance. A maximum EMI shielding effectiveness (SE) of 43.4 dB was achieved by composite foams, which is about twice that of a solid composite. Furthermore, as the supercritical CO_2_ foaming process reduces the density of the composite by 25–50%, the EMI SSE (specific shielding effectiveness)/t(thickness) of the composite reaches 402 dB/(g/cm^2^). Finally, compression tests were performed to show that the composites still maintained excellent mechanical properties after the supercritical CO_2_ foaming process (Figure 11c).

Foam composites with a three-dimensional network structure possess unique advantages in electromagnetic wave (EMW) dissipation owing to their internally interconnected skeletons and cell architecture. The multiple reflections and scatterings induced by multiple interfaces can effectively transform EMW energy into thermal energy, thereby significantly enhancing the EMI shielding performance [155]. Cui [156] fabricated a hierarchical structured foam of poly(vinylidene fluoride) (PVDF) modified with graphene nanosheets (GN) and nickel nanochains (Ni) (denoted as GN/Ni PVDF) using sc-CO_2_ foaming technology. The relationships between the microcellular structure of this material and its electrical conductivity and microwave absorption performance were systematically explored. The experiments demonstrated that after incorporating conductive and magnetic components, not only was the dielectric loss remarkably enhanced and the magnetic loss substantially improved, but also the high porosity characteristic greatly optimized the impedance matching of incident waves and facilitated multiple scatterings. The MXene foam prepared by Liu [157] exhibits hydrophobicity and excellent EMI shielding performance. With the introduction of a cell structure, its EMI shielding effectiveness (EM SE) reaches 70 dB, whereas the EM SE of the unformed sample is only 53 dB. In comparison with conventional two-dimensional solid structures, cell foam structures show superior shielding abilities due to the mechanisms of multiple reflections and scatterings of induced currents in the cell walls and support rods, as well as the dissipation of the electromagnetic wave field [158].

The matrix properties, filler type, and content of foam-structured composites also have a significant impact on their EMI attenuation behavior. In addition, the obtained shielding effectiveness (SE) has a strong correlation with thickness. The thicker the foam composite material, the higher the SE, the better the shielding effect on microwaves. So far, developing foam composites that are thinner, lighter, and have excellent performance remains a great challenge for further expanding their practical application potential [159,160,161]. Gao [162] prepared EP/rGO/Ni-chain microcellular composite foam materials, which exhibit good processability, excellent EMI shielding performance, and outstanding mechanical properties. Its maximum electrical conductivity is 0.44 S/m, EMI SE is 41.11 dB, compressive strength is 27.33 MPa, and density is 0.88 g/cm^3^, showing good application prospects as a high-performance EMI shielding material. Ma [163] prepared a layered foam/film structure by using PVDF/SiCnw/MXene (Ti_3_C_2_T_x_) composite foam as the absorbing layer and PVDF/MWCNT/GnPs composite film as the reflective layer, which effectively regulated the impedance matching and dissipation capacity of the absorbing layer.

Overall, the performance of polymer-based EMI shielding foams is not governed by a single parameter, but rather by the combined effects of electrical conductivity, density, cellular morphology, and mechanical support. Higher electrical conductivity generally indicates a more continuous conductive network and stronger electromagnetic dissipation; however, excessively high filler loading may suppress cell growth and increase processing difficulty. Reducing density and introducing a porous structure can improve impedance matching and promote multiple reflection and absorption, thereby enhancing the specific shielding effectiveness [164]. Nevertheless, excessive foaming may disrupt conductive pathways and lead to a decrease in absolute shielding effectiveness. With respect to cellular structure, a larger cell size and a higher specific surface area are generally favorable for enhanced absorption, whereas a smaller and more uniform cellular structure is more beneficial for maintaining conductive network continuity and mechanical integrity. Therefore, the fundamental design principle of EMI shielding foams is not merely to pursue higher conductivity or lower density, but to achieve a rational balance among shielding effectiveness, absorption-dominated shielding behavior, lightweight characteristics, and mechanical performance. To provide a clearer comparison, Table 1 summarizes representative information on polymer-based EMI shielding foams discussed in this review, including polymer type, filler composition, filler loading, EMI shielding effectiveness, and specific shielding effectiveness, where available [6,165,166].

Despite the substantial progress achieved so far, several important challenges remain for conductive microcellular foams in EMI shielding applications. In particular, the scalable and cost-effective manufacturing of high-performance foams is still limited, and recycling pathways for many conductive foamed systems have not yet been fully established. In addition, the coupled effects of thermal history, viscoelasticity, melt strength, filler dispersion, and phase morphology on bubble nucleation, growth, and stabilization are still not fully understood, especially in multiphase and multicomponent systems. It also remains challenging to simultaneously optimize conductivity, foaming ratio, cellular uniformity, mechanical robustness, and long-term shielding stability within a single material system. Future research should therefore place greater emphasis on establishing quantitative structure–process–property relationships, developing low-cost and green foaming strategies, improving recyclability and durability, and promoting multifunctional integration with sensing, thermal management, and intelligent response capabilities. With continued advances in supercritical foaming, hierarchical structure regulation, and conductive network engineering, conductive polymer microcellular foams are expected to show broader potential in lightweight electronics, aerospace systems, wearable devices, and other high-value applications [167,168,169].

### 5.2. Flexible Sensor

Most traditional sensors are made of metal or semiconductor materials, which inevitably have limitations in terms of flexibility, sensitivity, and complex processing technology [170,171]. To address these issues, researchers mix conductive fillers with an insulating polymer matrix through appropriate processing techniques to fabricate electrically conductive polymer composites (ECPC, Electrically Conductive Polymer Composites) [172,173]. These materials feature light weight, good flexibility, strong stability, and excellent mechanical properties, thus being widely applied in the field of flexible sensors [174]. For ECPC-based piezoresistive sensors, the sensing mechanism is generally attributed to stimulus-induced changes in the internal conductive pathways, including conductive-network rearrangement, contact variation, and changes in inter-filler distance, which lead to resistance responses [175]. Therefore, the rational design of the conducting network or conducting pathway is crucial for the sensing performance of ECPC-based sensors [176,177]. However, such sensors often suffer from poor repeatability in practical applications, mainly because conducting pathways are prone to crack propagation during repeated deformation, leading to the instability of the conducting network. To further improve sensor performance, especially sensitivity, lightweighting, and usability, designing sensors with cell structures is considered a promising strategy [178]. Currently, mainstream methods for introducing cell structures include non-foaming processes such as 3D printing [179], freeze drying [180,181,182], and dip coating [183,184,185], as well as conventional foaming technologies such as extrusion foaming [186] and injection foaming [187]. Among them, supercritical fluid batch foaming (SFBF) has received much attention in recent years due to its use of environmentally friendly physical blowing agents such as CO_2_/N_2_, controllable and stable cell structure, and low processing cost. sc-CO_2_ foaming can finely adjust the sensitivity of foam sensors by regulating the expansion rate of composite materials. The fabricated foam sensors possess not only high sensitivity and strong compressive resistance but also a wide operating range and are expected to meet the application requirements of next-generation intelligent sensors such as flexible electronics and wearable devices [188,189].

Multifunctional foam sensors are highly related to the types of polymer matrix and conductive fillers, as well as the porosity of the foamed material [190]. Foam sensors are suitable for various wearable applications and exhibit identifiable responsiveness to multiple environmental stimuli, including vibration, pressure, sound, temperature, humidity, and certain specific gases [191,192]. Moreover, such foam sensors can be applied as skin-adherent or non-contact tools for environmental monitoring, health management, and human motion detection [193,194]. Overall, compared with dense electrically conductive polymer composites, porous sensing materials not only possess lower density, but also exhibit a deformable microstructure that can more effectively amplify electromechanical responses. Their advantages are mainly reflected in three aspects. First, the reduced apparent modulus and enhanced compressibility contribute to improved responsiveness to subtle external stimuli. Second, the enlarged internal interfacial area and tunable conductive pathways facilitate more pronounced resistance responses through contact variation, disconnection/reconnection, and microcrack evolution. Third, porous structures increase the design flexibility of materials, enabling better integration of lightweight characteristics, mechanical flexibility, and sensing functionality into wearable devices. However, the enhancement in sensitivity is often accompanied by challenges in long-term stability, because highly deformable conductive pathways are more susceptible to irreversible damage under cyclic loading.

**Figure 12:**
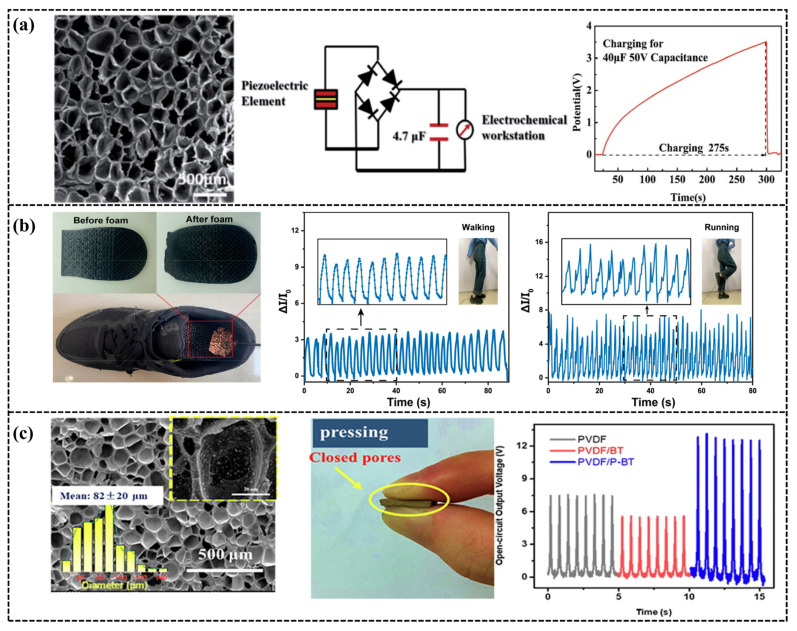
(**a**) The internal hole structure of the piezoelectric element and a 4.7 mF 50 V capacitor [199]. (**b**) TPU/4MWCNTs composite conductive foam as a plantar wearable sensor for gait recognition and resistance responses for different gait patterns: walking and running [197]. (**c**) Closed pore structure of PVDF-based composites and quantitative measurement of electrical signal output under compressive stimulation [200]. Under the CC BY-NC-ND 4.0 license (https://creativecommons.org/licenses/by-nc-nd/4.0/, accessed on 9 March 2026. No modifications were made).

Although porous flexible sensors are not limited to a single sensing mechanism, the representative systems discussed in this section can be broadly classified into two categories: piezoresistive and piezoelectric. Piezoresistive foams generally rely on conductive networks constructed from carbon nanotubes, graphene-derived fillers, or hybrid conductive pathways, and their response essentially originates from the dynamic evolution of conductive contacts during deformation. Previous studies have shown that the introduction of microcellular structures can significantly broaden the working strain range, improve compressibility, and enhance signal repeatability. Representative examples include PEBA/CNS composite foams, TPU/MWCNT composite foams, 3D-printed TPU-based foams, and RGO@Pebax composite foams [195,196,197,198]. For example. Yang [199] integrated selective laser sintering with supercritical carbon dioxide foaming to fabricate three-dimensional porous piezoelectric devices. The piezoelectric components produced through this method exhibit not only an increased β-crystalline content (the piezoelectrically active crystal form of PVDF) but also a reduced compressive modulus, making them suitable for applications in energy harvesters and smart sensors (Figure 12a). These studies collectively indicate that the performance improvement of piezoresistive porous sensors depends not only on the type of conductive filler, but also strongly on the uniformity of the pore structure, the recoverability of the conductive network, and the interfacial stability. However, such systems still generally face a trade-off between sensitivity and long-term cyclic stability. In contrast, the advantages of piezoelectric porous systems are more closely associated with the synergistic effects of structural compressibility and enhanced electromechanical coupling. For example. Guo [197] adopted the melt blending physical method to prepare conductive filaments using MWCNTs-modified thermoplastic polyurethane (TPU) as the raw material. Samples were prefabricated using an FFF printer and then saturated with CO_2_ in an autoclave before being removed and heated to foam. The composite foam effectively reduced residual strain, demonstrating the high resilience of the 3D-printed composite materials with a foam porous structure. The residual strain of the sample before foaming was >6% after a single cycle, and then gradually increased; the residual strain of the foamed samples is less than 5%. In addition, the composite foam has high sensitivity and can monitor subtle pressure changes. The sensing performance of the composite foam was evaluated, and the current signal remained stable under different loading rates and small compression strains. By using this highly resilient conductive composite material, a hierarchical shoe insole was designed that successfully detected human walking and running movements (Figure 12b). Guo [200] prepared a flexible poly (vinylidene fluoride-co-hexafluoropropylene) (PVDF)/barium titanate (BT) three-dimensional (3D) porous composite foam with improved interfacial compatibility and controlled porosity via supercritical carbon dioxide (sc-CO_2_) foaming technology and surface modification. By manipulating foaming conditions, PVDF foams with uniform cellular structures were successfully obtained. By the synergetic effect of BT and the modulated 3D porous structure, the piezoelectric output was significantly enhanced, with a peak output power density substantially higher than that of the flat film. Taking advantage of the comprehensive electromechanical performance, the integrated piezoelectric devices demonstrate applicability in fields such as transmission sensors for routine keyboard use (Figure 12c). Compared with piezoresistive systems, piezoelectric porous sensors are more attractive for self-powered sensing and energy-harvesting applications. However, their output stability, structural uniformity, and consistency in large-scale fabrication still require further optimization.

Overall, porous structures do not merely provide lightweight characteristics but also serve as an important structural parameter for regulating the performance of flexible sensors. According to the current literature, piezoresistive systems have been more extensively studied because of their relatively simple structural design and wide working range, whereas piezoelectric systems exhibit greater potential in self-powered sensing and energy-harvesting applications. Future research should place greater emphasis, while maintaining high sensitivity, on the controllability of pore structures, long-term cyclic stability, environmental adaptability, and the establishment of comparable evaluation methods across different systems.

### 5.3. Thermal Management

With the accelerating consumption of fossil fuels and the intensifying emission of greenhouse gases, global concern over energy consumption and environmental pollution continues to rise. In this context, energy conservation and emission reduction have become a key approach to alleviating this issue [201,202]. It is worth noting that heating and cooling energy consumption accounts for nearly half of global total energy consumption, making the development of insulation materials crucial for reducing carbon emissions and improving energy efficiency [203]. As a core medium in the process of energy conversion, storage. and transfer, thermal insulation materials directly achieve energy-saving goals by preventing heat transfer [204]. Compared with traditional intrinsically low-thermal-conductivity materials, polymer foams have been widely applied in fields such as automotive, transportation, and aerospace, owing to their multiple advantages, including being lightweight, easy to process, and highly designable [205,206].

The thermal insulation performance of polymer foams is closely related to their unique structure, which consists of a continuous polymer matrix phase and an enclosed gas phase [207]. The thermal conductivity of the enclosed gas phase is much lower than that of most polymer matrices, and so increasing the proportion of the gas phase has become the core strategy to reduce the overall thermal conductivity [208]. Among them, the cellular structure is a key parameter affecting the thermal insulation performance of foams; theoretically, increasing the porosity can significantly reduce the total thermal conductivity by reducing solid heat conduction paths and increasing the proportion of gas heat conduction [209,210,211]. Under this technical approach, the microcellular foaming technology using sc-CO_2_ as a physical blowing agent is regarded as an ideal method for preparing high-performance polymer foams due to its ability to precisely control the cellular structure [212,213]. However, although existing studies have successfully prepared foams with high cell density and small cell size through sc-CO_2_ foaming technology, the preparation of polymer foams with both ultra-high expansion ratio and ultra-low thermal conductivity still faces technical bottlenecks [214,215,216,217].

**Figure 13:**
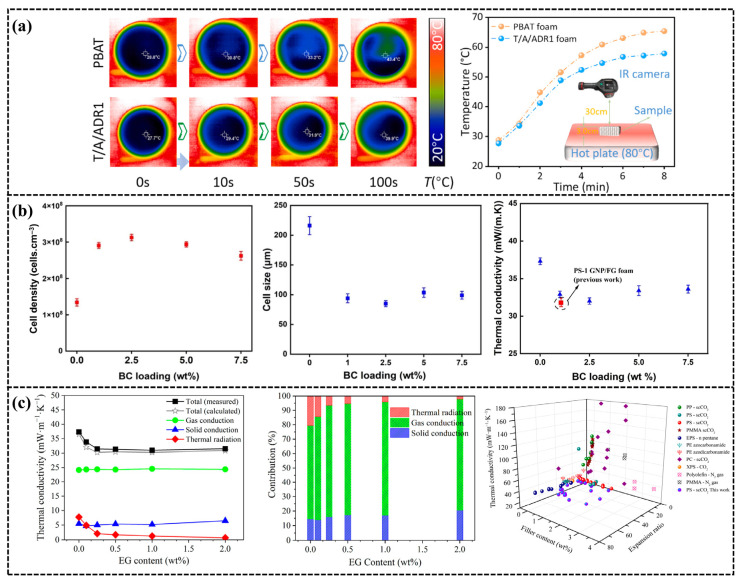
(**a**) Infrared thermal images with time of a hot plate at 80 °C and dynamic temperature variation [225]. (**b**) Average cell sizes, cell densities of PS–BC composite foams extruded using sc-CO_2_, variation in thermal conductivity of PS composite foams at different BC loadings [228]. (**c**) Analyzed thermal conductivity data of the PS/EG samples foamed in sc-CO_2_-pentane at 13.8 MPa. Total thermal conductivity of polymeric foams as a function of the carbonaceous additive content and the expansion ratio [230]. Under the CC BY-NC-ND 4.0 license (https://creativecommons.org/licenses/by-nc-nd/4.0/, accessed on 9 March 2026. No modifications were made).

In thermal insulation, polymer foams are one of the widely used materials, especially in building applications [218]. By reducing material usage and energy dissipation to improve energy efficiency, they have excellent economic and environmental benefits. When the cell structure size of the polymer is less than 1 μm, the Knudsen effect occurs, significantly reducing gaseous thermal conductivity (*λ*_g_) and radiative thermal conductivity (*λ*_r_). Current studies on the thermal management of polymer foams can be broadly categorized into three major strategies. The first strategy involves direct regulation of the cellular structure through optimization of the foaming process, including batch foaming, extrusion foaming, and injection foaming [219,220]. Existing studies have demonstrated that different foaming techniques can significantly reduce the thermal conductivity of neat polymer matrices. For example, XPS, PLA, PS, and PP foams all exhibit varying degrees of thermal conductivity reduction after foaming [221,222,223,224]. This indicates that the introduction of cellular structures alone can effectively disrupt solid-state heat conduction pathways, thereby serving as the fundamental origin of the thermal insulation performance of polymer foams. However, this strategy also has evident limitations. When higher expansion ratios are pursued, cell coalescence, structural instability, or foam shrinkage often occur, which in turn restricts the further achievement of ultralow thermal conductivity. The second strategy relies on modifying the blend, chain extension/crosslinking, or controlling crystallization to enhance foam stability and refine the cellular structure. The essence of this approach lies in improving melt strength, interfacial compatibility, and cell nucleation behavior, thereby maintaining a fine and stable cellular morphology under high-expansion conditions. For instance, Wang [225] found that, in the PBAT/PLA system, the incorporation of ADR strengthened interfacial interactions and suppressed foam shrinkage, thereby enabling lower thermal conductivity while maintaining a relatively high expansion ratio (Figure 13a). Similarly, Wang [226] found that, in the PLLA/PDLA system, the combination of HNT and stereocomplex crystal regulation facilitated the fabrication of composite foams with both high expansion ratios and low thermal conductivity. Overall, compared with process optimization alone, this strategy is superior in that it not only tailors the cellular morphology but also improves the thermodynamic and rheological stability during foam formation, making it more suitable for achieving the synergistic goal of “high expansion ratio-low thermal conductivity.” Nevertheless, its drawbacks should also be noted: the material design is more complicated, and the coupling mechanisms among material composition, interfacial interactions, and crystallization behavior still require further clarification. The third strategy introduces functional fillers to integrate thermal insulation with additional functionalities. In contrast to studies that focus solely on reducing thermal conductivity, this approach is increasingly oriented toward the practical requirements of devices and structural materials, while simultaneously considering mechanical reinforcement, electromagnetic interference shielding, ultralow dielectric properties, and sustainability. For example, Zhang [227] prepared PVDF/Ni-chain composite foams, which maintained relatively low thermal conductivity while improving electrical conductivity and mechanical strength, making them suitable for applications requiring both thermal insulation and electromagnetic functionality. Apurv Gaidhani [228] fabricated PS/BC composite foams through an sc-CO_2_ extrusion process, and the resulting materials demonstrated the synergistic potential of fillers in improving cellular structure, reducing thermal conductivity, and enhancing specific strength (Figure 13b). Shi [229] and Gong [230] further showed that the incorporation of functional components such as POSS and expanded graphite extended the application scope of foam materials toward low-dielectric, infrared-shielding, and superinsulating systems (Figure 13c). These findings suggest that the value of functional fillers lies not only in regulating heat transfer, but also in driving the evolution of polymer foams from single-function thermal insulation materials toward multifunctional thermal management materials. However, this route also involves clear trade-offs: although filler content and dispersion state can improve functionality, they may also compromise foaming stability, flexibility, and lightweight characteristics. In addition, the relevant studies on different thermal insulation materials discussed in this review are summarized in Table 2.

Overall, the enhancement of the thermal insulation performance of polymer foams is not governed by a single factor, but rather by the combined effects of cellular-structure regulation, matrix stabilization design, and the synergistic incorporation of functional components. Current studies have demonstrated that refined foaming strategies coupled with multicomponent design can effectively reduce thermal conductivity, and some systems have already approached the superinsulating regime. However, several common bottlenecks still remain in this field. First, achieving ultra-high expansion ratios and ultra-low thermal conductivity simultaneously is still challenging. Second, although finer cellular structures are beneficial for thermal insulation, they often come at the expense of increased processing complexity and higher cost. Third, the excellent thermal performance obtained at the laboratory scale has not yet been fully translated into long-term service stability and large-scale manufacturability. Therefore, future research should place greater emphasis on multiscale structural regulation mechanisms, the construction of highly efficient thermal barriers at low filler loadings, and the comprehensive balance among thermal insulation performance, mechanical reliability, and processability, thereby promoting the broader application of polymer foams in building energy conservation and advanced thermal management.

## 6. Conclusions

This paper focuses on conductive polymer microcellular foaming materials. Aiming to address the structural contradiction between the high rigidity and heavy weight of traditional conductive materials and the lightweight and multifunctional demands of emerging industries, systematic research is conducted from the aspects of material essence, preparation technology, and application scenarios, aiming to provide solutions for the innovative needs of fields such as aerospace and wearable devices.

Starting from the material essence, the research systematically explores the electrical conduction mechanism of conductive polymers, analyzes the influence of laws of factors such as molecular structure and filler dispersion on conductivity, and lays a theoretical foundation for the performance regulation of subsequent foaming modification. Aiming at the industry bottleneck of high electrical conductivity and low processability of conductive polymers, it reveals the dual optimization paths of microcellular foaming technology for material properties: through the dynamic process of polymer foaming induced by supercritical CO_2_, it not only enhances the uniformity of the conductive network and effectively reduces the material density but also imparts new functions such as electromagnetic interference shielding and flexible sensing through the construction of multiple interfaces. Meanwhile, in the field of thermal management, it breaks through the limitations of traditional materials and promotes the development of thermal management systems toward intelligentization and multifunctional integration through the multiscale design of the microcellular structure.

Despite the substantial progress achieved in recent years, several critical challenges still remain. First, the large-scale and cost-effective preparation of high-performance conductive polymer microcellular foaming materials is still limited, which restricts their practical application in industries. Second, the foaming behavior of conductive multiphase systems has not yet been fully understood, especially regarding the coupled effects of thermal history, viscoelasticity, melt strength, filler dispersion, and phase morphology on bubble nucleation, growth, and stabilization. Third, achieving synergistic optimization among conductivity, cellular structure, mechanical properties, and multifunctional performance remains a central challenge, particularly in complex systems with multiple components or hierarchical structures. In addition, issues related to recyclability, degradation behavior, sustainable foaming agents, and green manufacturing strategies also deserve greater attention in future research.

In the future, conductive polymer microcellular foaming materials are expected to develop toward multifunctional integration, intelligent regulation, precise structural design, and scalable green fabrication. Opportunities lie in aerospace components, wearable electronics, flexible sensors, and intelligent thermal management systems, where lightweight porous conductive structures may offer unique advantages. Therefore, future studies should not only focus on improving individual properties, but also on establishing quantitative structure–process–property relationships and promoting the transition of conductive polymer microcellular foaming materials from laboratory research to practical applications.

## Figures and Tables

**Table 1:** Comparative summary of polymer-based EMI shielding foams discussed in this review.

Polymer Foam	Filler Content	Filler Loading	EMI SE (dB)	Specific EMI SE (dB. g^−1^ cm^3^)	Ref.
PSU	CNTS	5 wt%	23.7	32.5	[146]
PVDF	CNT/SiCnw	4 wt% + 2 wt%	22	-	[147]
PBAT	Fe_3_O_4@_MWCNTs/Ni/Ag	15 wt%	68	-	[148]
PP	MXene/PANI	0.0449 vol% + 0.02 vol%	39.8	-	[149]
TPAE	SWCNT	2.5 wt%	46.1	209.8	[150]
EP	fMWCNTs/CTBN	5 wt% + 10 wt%	22.9	37.54	[152]
TPAE	Ti_3_C_2_T_x_ MXene	1.7 vol%	44	-	[153]
PVDF	CNTs/Ni	10 wt% + 10 wt%	43.4	80.4	[154]
EP	rGO/Ni	9.3 wt% + 0.46 wt%	41.11	50.13	[162]
PVDF	Upper: SiCnw + MXene Lower: MWCNTs + GnPs	30 wt% + 20 wt%	32.6	-	[163]

**Table 2:** Thermal insulation properties of different materials.

Material/System	Thermal Conductivity (mW·m^−1^·K^−1^)	Ref.
PLA/PLA-talc/PLA-clay foams	90	[222]
PS/MWCNT foams	32.8	[223]
PP/PTFE nano-fibrillar foams	36.5	[224]
PBAT/PLA/ADR foams	17.4	[225]
PLLA/PDLA/HNT foams	30.6	[226]
PVDF/Ni-chains foams	75	[227]
PS/BC foams	32	[228]
PS/EG foams	19.6	[230]

## Data Availability

No new data were created or analyzed in this study.
